# Diagnosis and antibiotic treatment of group a streptococcal pharyngitis in children in a primary care setting: impact of point-of-care polymerase chain reaction

**DOI:** 10.1186/s12887-019-1393-y

**Published:** 2019-01-16

**Authors:** Arundhati Rao, Bradley Berg, Theresa Quezada, Robert Fader, Kimberly Walker, Shaowu Tang, Ula Cowen, Dana Duncan, Joanna Sickler

**Affiliations:** 1 Molecular Genetics and Technical Pathology, Scott and White Medical Center–Temple, 2401 S. 31st Street, Temple, TX 76508 USA; 2 Baylor Scott & White Health, Round Rock, TX USA; 3 Roche Molecular Systems, Pleasanton, CA USA

**Keywords:** Cobas Liat strep a assay, Group a *Streptococcus*, Rapid antigen detection test, Molecular point-of-care testing

## Abstract

**Background:**

To compare the sensitivity and specificity of the recommended 2-step rapid antigen detection test (RADT) with confirmatory culture vs the point-of-care (POC) polymerase chain reaction (PCR) Roche cobas® Liat® Strep A test for detection of group A *Streptococcus* (GAS) in pediatric patients with pharyngitis, and to investigate the impact of these tests on antibiotic use in a large pediatric clinic.

**Methods:**

This prospective, open-label study was conducted at a single site during fall/winter 2016–2017. A total of 275 patients aged 3 to 18 years with symptoms of pharyngitis had a throat-swab specimen analyzed using RADT, POC PCR, and culture. The sensitivity, specificity, and percentage agreement (95% CI) between assays and a laboratory-based nucleic acid amplification test were calculated. DNA sequencing was used to adjudicate discrepancies. The RADT or POC PCR result was provided to clinicians on alternating weeks to compare the impact on antibiotic use.

**Results:**

A total of 255 samples were evaluated; 110 (43.1%) were GAS positive. Sensitivities (95% CI) for POC PCR, RADT, and culture were 95.5% (89.7–98.5%), 85.5% (77.5–1.5%), and 71.8% (62.4–80.0%), respectively. Specificities (95% CI) for POC PCR, RADT, and culture were 99.3% (96.2–99.98%), 93.7% (88.5–97.1%), and 100% (97.5–100%), respectively. Compared with RADT, POC PCR resulted in significantly greater appropriate antibiotic use (97.1% vs 87.5%; *P* = .0065).

**Conclusion:**

Under real-world conditions, RADT results were less specific and culture results were less sensitive than found in established literature and led to increased rates of inappropriate antibiotic use. POC PCR had high sensitivity and specificity and rapid turnaround times, and led to more appropriate antibiotic use.

**Trial registration:**

ID number ISRCTN84562679. Registered October 162,018, retrospectively registered.

## Background

Infection with *Streptococcus pyogenes* (group A beta-hemolytic streptococci; GAS) is the most common bacterial cause of acute pharyngitis and is responsible for an estimated 20–30% of sore throat cases in children [1, 2]. In the United States, the societal cost of GAS pharyngitis is estimated to range from $224 to $539 million per year, with children missing an average of 1.9 days of school/daycare and 42% of parents missing a mean of 1.8 days of work [3]. The timely initiation of antibiotics can effectively treat GAS pharyngitis, prevent the spread of infection to close contacts, reduce the duration of symptoms, and limit rare long-term complications [4]. Acute pharyngitis can be a result of either bacterial or viral pathogens and, as such, current clinical guidelines encourage the use of antibiotics only for confirmed cases of GAS[4]. Despite these recommendations, and the overall rates of documented GAS infections (5–30% of sore throat visits), antibiotics are often prescribed in as many as 60% of patient visits for sore throat [3, 5, 6]. Greater awareness of the development of bacterial resistance resulting from the overprescribing of antibiotics is increasing the urgency to move away from empiric antibiotic treatment for common respiratory infections.

The diagnosis of GAS pharyngitis based on clinical symptoms alone is not recommended [4]. The use of point-of-care (POC) rapid antigen detection tests (RADTs) to test throat-swab specimens obtained in the clinic can help diagnose GAS pharyngitis with the convenience of rapid turnaround times (e.g., < 10 min); [7] however, the overall sensitivity of these tests (70–90%) is low [8, 9]. Thus, current treatment guidelines for the diagnosis of acute pharyngitis in children recommend the use of RADTs of throat-swab specimens plus confirmatory bacterial culture in the case of negative results [4]. Although highly accurate (90–95% sensitivity) when performed correctly, [4] bacterial cultures are labor intensive and require additional infrastructure for sample transport to a separate laboratory and experienced staff to grow and test the bacteria – these factors can delay result reporting to the clinic by 48–72 h [10]. In addition, data have shown that the sensitivity of diagnostic culture conducted as part of routine clinical care is widely variable and lower than that observed with reference culture implemented in clinical trials [11, 12]. Despite clear recommendations for diagnostic testing to confirm GAS, a recent analysis of US claims data found that ≈ 25% of patients with pharyngitis receive no diagnostic testing and have high rates of empiric treatment (57.1%) [5].

In recent years, nucleic acid amplification tests (NAATs) have received approval from the US Food and Drug Administration (FDA) for diagnosing GAS pharyngitis, including several polymerase chain reaction (PCR) assays. The sensitivity and specificity of PCR assays are similar to those of bacterial reference culture, but PCR has better turnaround times – minutes to hours compared with days [12–14]. PCR has been implemented into pharyngitis treatment algorithms, as both a confirmatory method to replace culture and as a single test to eliminate the 2-step algorithm [15]. In 2015, the FDA approved the first Clinical Laboratory Improvement Amendments–waived PCR test for GAS for in-office POC use (cobas Strep A nucleic acid test for use on the cobas Liat System; Roche Molecular Systems, Pleasanton, CA); this system targets a segment of the *S. pyogenes* genome [16] to produce test results within 15 min in a clinic setting, offers sensitivity and specificity equivalent to and potentially improved over current diagnostic testing procedures, and eliminates the need for confirmatory culture [12, 15, 17].

The current study evaluated the feasibility of replacing the current standard of care (SOC) algorithm – the 2-step RADT with confirmatory culture – with POC PCR testing using the cobas Liat Strep A test. The clinical sensitivity, specificity, and the impact on patient care were prospectively evaluated at a large pediatric primary care clinic.

## Methods

This prospective, open-label study was conducted during September 2016 to January 2017 at a single large primary pediatric care clinic within the Baylor Scott and White integrated system located in the suburbs of Austin, TX (Scott and White Round Rock Clinic, 7 providers, 100 to 150 patients per day). Pediatric patients aged 3–18 years with clinical signs and symptoms of GAS pharyngitis –defined as the presence of sore throat and ≥ 1 other symptom (redness of the posterior pharyngeal wall, pharyngeal or tonsillar exudate, tonsillar swelling, tender cervical lymphadenopathy, and/or fever > 100 °F) – were eligible for inclusion in the study. Subjects treated with antibiotics currently or within the previous 7 days were excluded. All subjects or their guardians provided written informed consent to participate in the study, and the study protocol and informed consent form were approved by the Baylor Scott and White Institutional Review Board (Temple, TX) prior to study initiation.

### Comparison of POC RADT, bacterial culture, and POC PCR

Performance of the QuickVue Strep A test (Quidel RADT) manufactured by Quidel Corporation (San Diego, CA, USA), bacterial culture, and cobas Liat Strep A test (POC PCR) manufactured by Roche Diagnostics (Pleasanton, CA, USA) were evaluated by using the laboratory-based Solana GAS NAAT manufactured by Quidel Corporation (San Diego, CA, USA) as the reference method. All discordant results between the 4 methods were adjudicated with bidirectional sequencing. The sequencing primers were adapted from Kaltwasser et al [18]. Final results were based on the NAAT and sequencing results for discordant samples.

At the time of visit, 2 throat-swab specimens were collected using the Copan Liquid Amies Elution Swab (ESwab) Collection and Transport System. Swab 1 was used for the RADT; swab 2 was used for POC PCR testing at the outpatient laboratory within the clinic, conducted by the on-site staff and study coordinator. After POC PCR testing, swab 2 was sent to an offsite laboratory (Scott and White Medical Center, BSW Health, Temple, TX) for culture (by transferring the suspension of patient sample in transport medium per the manufacturer instructions for use). Throat swabs were plated onto 5% sheep blood agar (BD Microbiology Systems, Sparks, MD) and incubated at 35 °C in 5% CO_2_ for up to 48 h. Suspicious beta-hemolytic colonies were typed either directly or subcultured to obtain isolated colonies. Lancefield typing for Groups A, C and G was performed by using the PathoDX Strep group typing kit (REMEL, Lexena, KS) according to the manufacturer’s instructions.

Samples were subsequently frozen. Laboratory-based NAATs and sequencing were conducted on the thawed specimens later that same winter.

The overall sensitivity, specificity, and percentage agreement between the different testing methods and the respective 95% CIs (via Clopper–Pearson, exact) were calculated. A minimum enrollment requirement of 60 patients positive for GAS, whereby the lower 95% CI for sensitivity and specificity would be > 90%, was estimated. This cutoff was based on the reported 98.3% sensitivity and 94.2% specificity of the cobas Liat Strep A test [16] and the 97.1% sensitivity and 60.5% specificity of the QuickVue Strep A test (clinical site validation study; data on file) in order to demonstrate a statistically significant performance difference between the 2 tests.

Samples with discordant results were re-tested using the cobas Liat Strep A system because the additional pre-analytic steps (2 vortexing steps and 1 incubation step) required for the reference method increase the likelihood of GAS being detected when low levels of bacteria were present. Low-positive results were monitored by noting the PCR cycle threshold values. Samples with discordant results between the initial cobas Liat Strep A test and the retest were analyzed separately. Bacterial culture was originally defined as the reference method for comparison of the assays; however, due to the unexpectedly low sensitivity rates observed during the study, routine laboratory NAATs were initiated for all samples in addition to culture.

### Evaluation of clinical management of GAS using different assays

Although both the POC PCR and RADT assays were performed in real time within the clinic, only 1 assay result was provided to the clinician for patient management—these results were alternated on a weekly basis throughout the study period. Details of the clinical management of the patient based on clinical judgement and the results of the POC test were documented and subsequently collected from the electronic medical record for the entire care episode; differences in antibiotic prescribing (treatment changes, additional tests ordered, hospital admissions, and follow-up appointments) were compared between POC testing methods. The appropriate use of antibiotics was defined as either a final positive GAS result plus initiation of antibiotics or a final negative GAS result and no antibiotics prescribed; all other uses were considered inappropriate. The proportions of appropriate antibiotic use between the 2 management arms (POC PCR and POC RADT plus confirmatory culture) were compared using Fisher’s exact test at a significance level of 0.05.

## Results

**Fig. 1:**
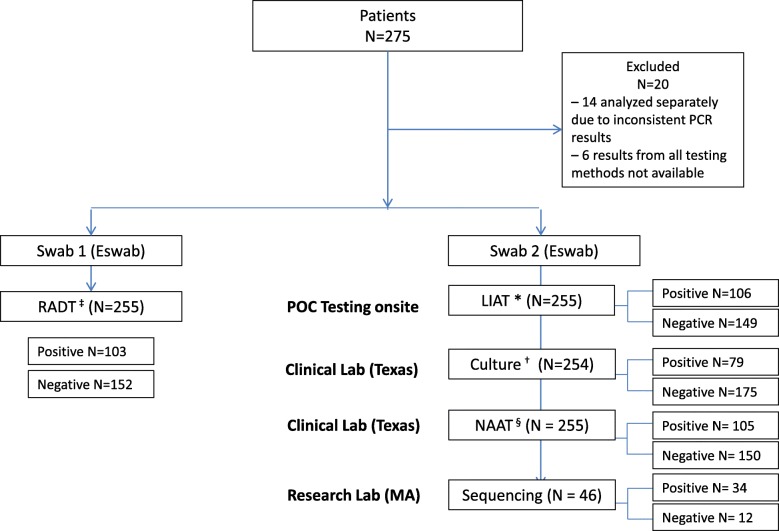
Sampling methods and analysis of the different tests for GAS

A total of 275 samples were collected, of which 255 (92.7%) were evaluable for performance testing; 14 of 275 (5.1%) samples were analyzed separately due to inconsistent POC PCR results, and results from all testing methods were not available for 6 of 275 (2.2%) samples. During the study period, 152 (59.6%) and 103 (40.4%) patients had POC RADT and POC PCR assay results, respectively, that were available to the clinician at the time of clinical assessment (Fig. 1). A total of 110 samples were determined to be positive for GAS, with an overall incidence of 43.1%: 46.1% (70/152) in the RADT arm and 38.8% (40/103) in the POC PCR arm.

### Comparison of RADT, bacterial culture, and POC PCR results

A total of 105 positive POC PCR results were found to be true-positive for GAS and 144 POC PCR results were found to be true negative for GAS, for an overall sensitivity (95% CI) of 95.5% (89.7–98.5%; Table 1). In contrast, sensitivity rates (95% CI) of 85.5% (77.5–91.5%) and 71.8% (62.4–80.0%) were observed with RADT and bacterial culture, respectively (Table 1). In particular, culture produced 31 false-negative results that were subsequently determined to be positive for GAS by NAAT. Overall, the specificity of each testing method was high, ranging from 93.7% for RADT to 99.3% for POC PCR to 100% with culture. Although not statistically significant, the RADT test had 9 false-positive results. The POC PCR test had a statistically significant greater overall percentage agreement of 97.6% (94.9–99.1%) than either the RADT (90.2%; 85.9–93.6%) or bacterial culture (87.8%; 83.1–91.6%) tests (Table 1).

**Table 1:** Clinical performance of POC PCR, laboratory PCR, bacterial culture, and POC RADT when compared with final results by sequencing for group A *Streptococcus* (*n* = 255)

	Cobas Liat POC PCR^a^			Quidel QuickVue POC RADT			Bacterial culture		
Final result^b^	Positive	Negative	Total	Positive	Negative	Total	Positive	Negative	Total
Positive	105	1	106	94	9	103	79	0	79
Negative	5	144	149	16	136	152	31	144	175
Total	110	145	255	110	145	255	110	144	254
Sensitivity n/N (%, 95 CI)	105/110 (95.5%, 89.7–98.5)			94/110 (85.5%, 77.5–91.5)			79/110 (71.8%, 62.4–80.0)		
Specificity n/N (%, 95 CI)	144/145 (99.3%, 96.2–99.9)			136/145 (93.7%, 88.5–97.1)			144/144 (100.0%, 97.5–100.0)		
PPV n/N (%, 95 CI)	105/106 (99.1%, 94.9–99.9)			94/103 (91.3%, 84.1–95.9)			79/79 (100.0%, 95.4–100.0)		
NPV n/N (%, 95 CI)	144/149 (96.6%, 92.3–98.9)			136/152 (89.5%, 83.5–93.9)			144/175 (82.3%, 75.8–87.6)		
OPA n/N (%, 95 CI)	249/255 (97.6%, 94.9–99.1)			230/255 (90.2%, 85.9–93.6)			223/254 (87.8%, 83.1–91.6)		

*NPV* negative predictive value, *OPA* overall percentage agreement, *PPV* positive predictive value

^a^cobas Liat Strep A (POC) and Solana GAS NAAT (laboratory based). PCR via Clopper–Pearson (exact)

^b^Results based on concordant test results or bidirectional DNA sequencing when results were discordant

### Evaluation of clinical management of GAS using different assays

**Fig. 2:**
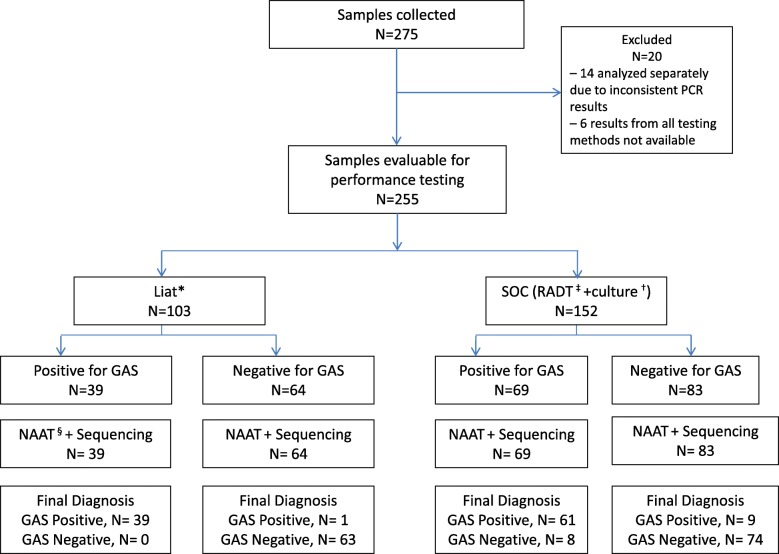
Clinical impact analysis using the Liat PCR test compared with RADT and culture

Culture results took a median of 2 days (mean, 1.9 days; range, 1–3 days) from the time of sampling, and positive results were reported ≈ 1 day earlier than negative results (median, 1–2 days vs 2–3 days). In general, POC PCR results took 5–10 min longer than results obtained with RADT. The test results for each arm and the final GAS results are shown in Fig. 2. The use of POC PCR resulted in the appropriate use of antibiotics in 97.1% of cases compared with 87.5% of cases for the standard of care (SOC), RADT plus confirmatory bacterial culture (*P* = .0065 by Fisher’s exact test; Table 2). The use of SOC in this population resulted in 9 patients with positive results not receiving treatment with antibiotics and 10 with negative results receiving treatment. The use of the SOC or POC PCR testing had no statistically significant impact on rates of additional patient follow-up visits (data not shown). No hospital admissions occurred in the study.

**Table 2:** Appropriate antibiotic prescribing in relation to group A Streptococcal testing results

Antibiotic use		Final result*			
		SOC^a^ (*n* = 152)		Liat^b^ (*n* = 103)	
		Positive	Negative	Positive	Negative
Antibiotic	Yes	61	10	38	1
	No	9	72	2	62
Appropriate antibiotic use, % (n/N)^c^		87.5 (133/152)		97.1 (100/103)	

*Final result by bidirectional DNA sequencing; *P* = .0065

^a^RADT plus culture

^b^cobas Liat Strep A POC PCR assay

^c^Appropriate antibiotic use defined as follows: final result positive plus antibiotics = yes or final result negative plus antibiotics = no. SOC % = (61 + 72)/ (61 + 10 + 9 + 72); Liat% = (38 + 62)/(38 + 1 + 2 + 6 + 62)

Thirteen (4.7%) negative POC PCR results were positive when re-tested with POC PCR after undergoing a freeze/thaw and pre-analytic steps needed for NAAT. These specimens had high cycle threshold values indicating low bacterial loads. The original POC PCR was conducted on the same day as sample collection and generally minutes after collection. Five of these patients received antibiotics. None of these patients required follow-up visits or prescription changes after the initial visit. One (0.4%) positive POC PCR result was negative on the re-test. This patient did not receive antibiotics.

## Discussion

Visits to healthcare providers for a sore throat are common in the United States and lead to an antibiotic prescription in upwards of 60% of cases [5, 6]. From 2010 to 2011, pharyngitis was the third most common infection in which antibiotics were prescribed, and an estimated 34% of patients aged < 19 years received an inappropriate antibiotic prescription [15]. The current GAS diagnostic algorithm is based on data demonstrating that RADTs when utilized in routine clinical care are specific but not sensitive [8] and bacterial cultures are both highly specific and sensitive [4]. In theory, this 2-step process would allow clinicians to have confidence when applying the GAS diagnostic algorithm in clinical practice.

Our results show that RADT testing had both decreased sensitivity (85.5% [77.5–91.5%]) and lower than expected specificity (93.7% [88.5–97.1%]). These results were not statistically significant but the difference in overall level of agreement was (97.6% [94.9–99.1%] for POC PCR compared with 90.2% [85.9–93.6%] for RADT) indicating a clear trend. These results are in agreement with the findings of 2 recent meta-analyses of RADTs for GAS in children that found a pooled sensitivity and specificity of 86% (79–92%), 87% (84–89%) and 92% (88–95%), 96% (95–97%), respectively [7, 19]. Similar findings were obtained in a study looking at the performance of the Quidel RADT in routine clinical use [20]. These findings are consistent with the sensitivity reported in the package insert of 95% (86–96%) [21]. Our data also showed that bacterial culture had much lower sensitivity rates than expected; [4] overall rates were lower than those observed with RADT (71.8% vs 85.5%; Table 1). Thus, a clinician’s reliance on the current SOC testing algorithm could lead to misdiagnosis of GAS pharyngitis and missed or inappropriate antibiotic use. This observation was confirmed when clinical management was included in the analysis; appropriate antibiotic use was more common with the single-step POC PCR test than with the 2-step RADT with reflex culture (97.1% vs 87.5%; *P* = .0065; Table 2).

Historically, bacterial culture is considered the gold standard because it is believed to be both highly sensitive and highly specific. In clinical trials, cultures are generally performed at a central reference laboratory under rigorous procedural oversight and optimized transport conditions to prioritize sensitivity, with limited concern about turnaround times. The sensitivity results in this study (71.8% [62.4–80.0%]) and others (81 and 86.8%) that look at culture [11, 12] under real-world conditions, often with less rigorous procedural oversight, raise questions about its use in routine clinical care, especially in light of the time delay to report results back to providers.

Turnaround time is a high priority for patient care, timely results are necessary for optimal management. This is particularly important for streptococcal pharyngitis, because children are highly contagious until treated and both individual and community health are adversely affected by delays in diagnosis. Parental desire for a timely diagnosis and treatment, along with the additional resources needed to follow-up on culture results and report these results to parents, place additional strains on providers. As a result, clinicians often empirically prescribe antibiotics before receiving the final test results, or treat based on clinical presentation alone, which has low proven specificity [2]. Prudent use of antibiotics by outpatients is necessary to prevent the development and spread of bacterial resistance [22].

POC PCR can provide highly accurate results for patients at the time of the office visit, eliminating the need for follow-up confirmatory testing of negative results and for empiric treatment. Providers in this busy pediatric clinic believed that the increased wait time and decreased room availability were offset by more appropriate antibiotic use, the potential for fewer missed days of school/work for patients and their parents, and less staff and provider time and resources while awaiting confirmatory culture results. In addition, workflow efficiencies can also be realized by minimizing the need for patient follow-up after laboratory results are received days later, even more important in these days of limited operating processes.

Studies support a 15–20% rate of *S. pyogenes* carriage among asymptomatic school aged children and 25% among asymptomatic household contacts of children [23]. A potential limitation of PCR is that the high sensitivity will increase detection of GAS colonization in patients who do not require treatment. In this study, 13 patients were negative for GAS by POC PCR; however, GAS was detected in these samples after further pre-analytic steps allowing for continued bacterial replication before laboratory NAAT testing was performed. High PCR cycle threshold values confirm these samples had low bacterial loads. Evaluation of clinical management data indicated no negative consequences for these patients, who did not receive antibiotic treatment.

In the context of the physician office, utilizing a more sensitive POC PCR method has the potential advantage to overcome poor sampling and identify acute cases of GAS. However, samples transported to the lab for testing and undergoing additional pre-analytic steps allowing for continued bacterial replication may exacerbate the potential to identify colonized patients. Further study of this issue is needed and it highlights the importance of careful patient selection for testing based on Centor score criteria including sore throat and tonsil or pharynx inflammation to minimize the potential for over-diagnosis through identification of colonized patients.

Performing nucleic acid amplification outside of the laboratory setting raises concerns about potential for contamination and false-positive results. Even when indicated for use in these settings, considerations should be given to system design and potential for contamination from improper use. The system used in this study utilizes a closed sealed tube, never opened after the sample was inserted, which in theory limits potential for amplicon contamination. Training of staff is critical to ensure they leave tubes sealed, follow recommended disposal procedures, and understand the increased contamination risks.

Cost pressures on primary care providers continue to increase as reimbursement decreases and payers increasingly value cost-effective considerations when making reimbursement decisions. NAAT and therefore PCR accuracy provides increased value to clinical care and health systems. As a result it is reimbursed at a higher rate compared to RADT. In the US for example, fee for service rates are $43.33 for NAAT, $16.81 for RADT and $8.18 for culture. The ability to provide patients and providers with definitive results at the time of the clinic visit, without the need for a culture and the resultant delay in confirmatory results or inappropriate antibiotic use relying on RADT, should provide a direct cost savings to both clinics and patients/parents. These benefits could also provide savings for health systems reliant on fixed reimbursement amounts for a healthcare episode such as accountable care organizations in the US. Additional pharmacoeconomic research could help to show these benefits through direct costing and health analyses.

## Conclusion

In conclusion, the use of the POC PCR cobas Liat Strep A test in a large pediatric clinic increased the rates of accurately diagnosing GAS pharyngitis, provided results at the time of the clinic visit, and reduced inappropriate antibiotic prescribing compared with the recommended current treatment paradigm of RADT plus reflex culture. The American Society for Microbiology has recommended that clinics redesign their office workflow to incorporate POC testing [24]. Additionally, PCR-based GAS pharyngitis testing, while not currently recommended in treatment guidelines, may produce results on par with gold standard laboratory testing; incorporation of PCR-based methods into practice guidelines could inform providers of the benefits of these methods and encourage their use [24]. Thus, the use of POC PCR to diagnose GAS pharyngitis should be considered to improve patient care and office/provider efficiency.

## Abbreviations

GAS: Group A Streptococcus
NA: Not available
NAAT: Nucleic acid amplification test
NP: Not performed
NPV: Negative predictive value
OPA: Overall percentage agreement
PCR: Polymerase chain reaction
POC: Point of care
PPV: Positive predictive value
RADT: Rapid antigen detection test
SOC: Standard of care
